# Quality of care in the context of universal health coverage: a scoping review

**DOI:** 10.1186/s12961-022-00957-5

**Published:** 2023-03-23

**Authors:** Bernice Yanful, Abirami Kirubarajan, Dominika Bhatia, Sujata Mishra, Sara Allin, Erica Di Ruggiero

**Affiliations:** 1 Division of Social and Behavioural Health Sciences, Dalla Lana School of Public Health, University of Toronto, Toronto, Canada; 2 Institute of Health Policy, Management and Evaluation, Dalla Lana School of Public Health, University of Toronto, Toronto, Canada; 3 Centre for Global Health, Dalla Lana School of Public Health, University of Toronto, Toronto, Canada

**Keywords:** Quality of care, Universal health coverage, Health systems, Equity

## Abstract

**Introduction:**

Universal health coverage (UHC) is an emerging priority of health systems worldwide and central to Sustainable Development Goal 3 (target 3.8). Critical to the achievement of UHC, is quality of care. However, current evidence suggests that quality of care is suboptimal, particularly in low- and middle-income countries. The primary objective of this scoping review was to summarize the existing conceptual and empirical literature on quality of care within the context of UHC and identify knowledge gaps.

**Methods:**

We conducted a scoping review using the Arksey and O’Malley framework and further elaborated by Levac et al. and applied the Preferred Reporting Items for Systematic Reviews and Meta-Analyses (PRISMA) Extension for Scoping Reviews reporting guidelines. We systematically searched MEDLINE, EMBASE, CINAHL-Plus, PAIS Index, ProQuest and PsycINFO for reviews published between 1 January 1995 and 27 September 2021. Reviews were eligible for inclusion if the article had a central focus on UHC and discussed quality of care. We did not apply any country-based restrictions. All screening, data extraction and analyses were completed by two reviewers.

**Results:**

Of the 4128 database results, we included 45 studies that met the eligibility criteria, spanning multiple geographic regions. We synthesized and analysed our findings according to Kruk et al.’s conceptual framework for high-quality systems, including foundations, processes of care and quality impacts. Discussions of governance in relation to quality of care were discussed in a high number of studies. Studies that explored the efficiency of health systems and services were also highly represented in the included reviews. In contrast, we found that limited information was reported on health outcomes in relation to quality of care within the context of UHC. In addition, there was a global lack of evidence on measures of quality of care related to UHC, particularly country-specific measures and measures related to equity.

**Conclusion:**

There is growing evidence on the relationship between quality of care and UHC, especially related to the governance and efficiency of healthcare services and systems. However, several knowledge gaps remain, particularly related to monitoring and evaluation, including of equity. Further research, evaluation and monitoring frameworks are required to strengthen the existing evidence base to improve UHC.

## Background

According to the World Health Organization, universal health coverage (UHC) is achieved when ‘all people and communities can use the promotive, preventive, curative, rehabilitative and palliative health services they need, of sufficient quality to be effective, while also ensuring that the use of these services does not expose the user to financial hardship’ [1]. UHC has gained renewed attention from researchers and policymakers following its inclusion in the 2030 Agenda for Sustainable Development (SDGs). SDG target 3.8 calls for achieving ‘universal health coverage, including financial risk protection, access to quality essential healthcare services and access to safe, effective, quality and affordable essential medicines and vaccines for all’ [2].

While there is growing evidence linking UHC to different health, economic and social outcomes, recent estimates suggest that about 800 million people globally still do not have access to full financial coverage of essential health services, including but not limited to high-income countries [3]. The WHO’s well-established UHC cube identifies three dimensions of UHC: (1) population (who is covered); (2) services (services that are covered); (3) direct costs (the proportion of the costs that are covered) [4]. Absent from the cube is the explicit inclusion of quality of care. However, without attention to the quality of care provided, increasing service coverage alone is unlikely to produce better health outcomes. As such, quality of care is critical to the achievement of UHC. A high-quality health system has been defined as one ‘that optimises health care in a given context by consistently delivering care that improves or maintains health outcomes, by being valued and trusted by all people, and by responding to changing population needs’ [5, p. e1200].

Current evidence suggests that quality of care is suboptimal, particularly in low- and middle-income countries (LMICs) [6]. While the era of the Millennium Development Goals (MDGs) expanded access to essential health services in LMICs, poor quality of care remains a significant problem, and explains persistently high levels of maternal and child mortality [6]. In addition, poor quality of care is estimated to cause between 5.7 and 8.4 million deaths yearly in LMICs [7]. Low-quality services are also an issue in high-income countries (HICs), particularly for disadvantaged populations such as immigrant and Indigenous groups [6, 8].

As such, efforts to achieve UHC focused solely on expanding access to care are insufficient. Achieving UHC will require a more deliberate focus on quality of care across its various dimensions including effectiveness, safety, people-centredness, timeliness, equity, integration of care and efficiency [6]. However, existing literature synthesizing evidence on the quality of care within the context of UHC is more limited.

### Objective

The primary objective of this scoping review is to synthesize and analyse the existing conceptual and empirical literature on quality of care within the context of UHC. The secondary objective is to identify knowledge gaps on quality of care within the context of advancing UHC and highlight areas for further inquiry.

### Methods

We conducted a scoping review using the five-stage scoping review framework proposed by Arksey and O’Malley [9] and further elaborated by Levac et al. with the following stages [10]: (1) formulating the research question; (2) searching for relevant studies; (3) selection of eligible studies; (4) data extraction and (5) analysing and describing the results. In addition, we followed the Preferred Reporting Items for Systematic Reviews and Meta-Analyses (PRISMA) Extension for Scoping Reviews reporting guidelines [11]. In accordance with the guidelines, our protocol is publicly available through Open Science Forum [12]. The scoping review methodology was selected due to its relevance to both identifying emerging and established content areas, and integration of diverse study methodologies [13]. As such, our methodology was well-aligned with the exploratory aims of our study.

To synthesize the existing knowledge on quality of care within the context of UHC, we focused on retrieving and analysing relevant reviews (as opposed to primary research studies). Bennett et al. [14] applied this overview of reviews approach in identifying health policy and system research priorities for the SDGs.

#### Information sources and search strategy

We developed the search strategy in consultation with a research librarian with expertise in public health and health systems. After finalizing our search in MEDLINE (Ovid) through an iterative process involving pilot tests, we completed a systematic search of MEDLINE (Ovid), EMBASE (Ovid), CINAHL-Plus (EBSCO), PAIS Index, ProQuest and PsycINFO (Ovid) for articles published from 1 January 1995 to 27 September 2021. The date cut-off of 1995 was selected to capture articles published during the period leading up to the adoption of the MDGs. We applied adapted search filters from the InterTASC Information Specialists’ Subgroup Search Filter Resource for each database [15].

**Fig. 1:**
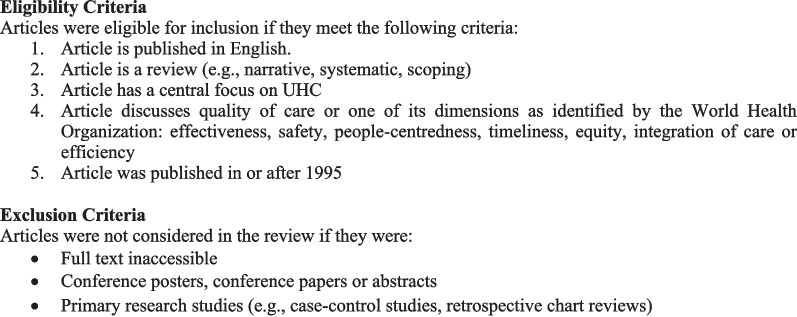
Eligibility and exclusion criteria

Our searches combined terms related to the concepts of (1) UHC (e.g. universal health insurance, universal coverage) and (2) quality of care and its seven dimensions (e.g. equity, safety, people-centredness). Our search strategy is available in Appendix A. Figure 1 outlines the eligibility criteria we used to assess studies for inclusion in the review.

#### Data management

Results from database searches were managed through Covidence (www.covidence.org) for deduplication and screening.

#### Study selection

Two reviewers (BY&AK) independently assessed studies against the eligibility criteria in two phases: (1) titles and abstracts and (2) full-text articles. A pilot test of the title and abstract screening was completed for approximately the first 100 search results. The two reviewers discussed disagreements to revise eligibility criteria as required. Any disagreements were resolved via consensus and in consultation with senior co-authors.

#### Data extraction

BY & AK independently completed data extraction for the first 10 articles using a standardized form. Following the pilot, the full data extraction was completed by the two reviewers in parallel. We extracted data on key study characteristics and according to each domain and subcomponent identified in Kruk et al.’s [5] framework described in the following section. The process of data extraction was iterative, with the form subject to revisions. Geographic regions were classified either by WHO regions [16] or through self-identification by the articles, such as a global focus, LMICs, HICs, ‘developing’ or ‘developed’.

#### Data synthesis

We synthesized the results through both a descriptive summary and a qualitative, narrative synthesis. We anchored our narrative synthesis in Kruk et al.’s [﻿^5^] conceptual framework for high-quality health systems. The framework draws from Donabedian’s well-known conceptual model of quality of care, which was first developed in the 1960s and identifies structures, processes and outcomes as three components of quality of care. Kruk et al. [5] offer a new evidence-based framework relevant to present-day health systems, recognizing the heterogeneity of health systems across HIC and LMIC contexts.

They define three key domains of a high-quality health system, which they argue should be at the core of implementing and advancing UHC: foundations, processes of care and quality impacts. Foundations refer to the context and resources required to lead a high-quality health system. Processes of care include competent care and systems, relating to evidence-based effective care and health systems’ ability to respond to patient needs. Quality impacts include both patient and provider-reported health outcomes and client confidence in the health system, as well as economic benefits such as a reduction of resource waste and financial risk protection. The Kruk et al. [5] framework does not explicitly address equity; however, the authors state that equity in the quality of healthcare is critical, which they define as ‘the absence of disparities in the quality of health services between individuals and groups with different levels of underlying social disadvantage [p. e1214].’ When compared with Donabedian’s model for evaluating the quality of care [17], Kruk et al. [5] offer a much more elaborated framework that explicitly names a range of subcomponents to guide quality measurement and improvement (e.g. governance, positive user experience, etc.).

As our scoping review examines the existing literature on quality of care within the context of UHC and identifies knowledge gaps, Kruk et al.’s [5] framework provided a useful analytic tool by which to organize and interpret our findings.

We organized the results from our narrative synthesis according to each component of the framework (foundations, processes of care and quality impacts), addressing equity as a cross-cutting theme across these components. Table 1 summarizes the components and subcomponents of the framework.

**Table 1:** Summary of Kruk et al.’s conceptual framework on high-quality health systems

Components	Subcomponents	Abridged description	Example
Foundations	Population	Individuals, families and communities; system users; health literacy and cultural norms	Health literacy of vulnerable populations
	Governance	Leadership structures including contracting, payment and institutions for accountability; institutions for measurement, evaluation and improvement; trustworthy data	Transparent audits to prevent corruption
	Platforms	The accessibility and organization of care delivery, including geographic access and distribution of facilities	Public and private mix of healthcare financing and delivery
	Workforce	Personnel-based resources within the health system, including healthcare workers and managers	Delegation of roles and task-shifting
	Tools	Physical and technological resources including software, equipment, medical supplies and use of data	Integration of electronic medical records
Processes of care	Competent care and systems	Evidence-based healthcare that provides correct and appropriate diagnosis and treatment	Accurate screening and diagnosis of non-communicable diseases
	Positive user experience	People-centered care that involves patient values, including respect, choice of provider, wait times and ease of use	Patient satisfaction with wait times
Quality impacts	Better health	Effects on patient symptoms, health status, function, quality of life, morbidity and mortality	Maternal and child mortality rates
	Confidence in system	Patient-reported satisfaction and trust in health systems	Voluntary re-enrollment in insurance schemes
	Economic benefit	Ability to participate in the economy, financial protection, and reduction of financial and resource waste	Reduction in unnecessary healthcare

## Results

### Description of included reviews

The database searches yielded 4128 results after deduplication. Following screening, 45 articles that met eligibility criteria were included in the review. The search results are shown in Appendix A and a summary of each article is presented in Table 2. Narrative reviews comprised 40.0% of the studies (*n* = 18), 35.6% were systematic reviews (n = 16), while 20.0% were scoping reviews (*n* = 9), and 4.4% were overviews of systematic reviews (*n* = 2). Of the 45 reviews, 28 covered multiple WHO regions (62.2%). This included reviews with a broad global focus, reviews focused on LMICs, ‘developing’ or ‘developed’ countries, as well as reviews with an explicit focus on more than one of six WHO regions. Regarding the dimensions of quality of care, equity was the most well represented, examined by 40 of the studies (88.9%). Integration of care and safety were the least represented across the studies, each examined by 11 of the reviews (24.4%). We did not formally appraise the quality of studies included in our review, which is not required for a scoping review given its overarching aim to map the scope and size of the available literature on a given topic.

**Table 2:** Description of included studies

Authors, year	Title	Study design	Geographic regions	Quality dimensions	Review objectives	Key findings
Agarwal et al., 2019	A conceptual framework for measuring community health workforce performance within primary health care systems	Narrative review	LMICs	Effectiveness, equity	Identify indicators to monitor community health workers’ performance in LMICs	Identified 21 subdomains to measure CHW performance including service quality and CHW absenteeism and attrition
Alhassan et al., 2016	A review of the National Health Insurance Scheme in Ghana: what are the sustainability threats and prospects?	Scoping review	AFR	Effectiveness, people centredness, timeliness, equity	Describe threats to, and opportunities to strengthen the sustainability of the NHIS in Ghana	Poor perceived quality of care within Ghana’s NHIS has reduced clients’ trust in the insurance scheme and decreased re-enrollment rates
Almeida, 2017	The role of private non-profit healthcare organizations in NHS systems: implications for the Portuguese hospital devolution programme	Narrative review	EUR	Effectiveness, people centredness, efficiency	Evaluate the effects of privatization on the efficiency, quality and responsiveness of services in publicly available universal health care systems	Results suggest that privatization, through transferring management of some hospitals from the public sector to private, non-profit organizations can improve efficiency and access within NHS systems without sacrificing quality
Ansu-Mensah et al., 2020	Maternal perceptions of the quality of care in the Free Maternal Care Policy in sub-Sahara Africa: a systematic scoping review	Scoping review	AFR	Integrated care, people centredness, timeliness	To summarize evidence on the perceptions of the quality of free maternal healthcare services in sub-Saharan Africa	8 of 13 included studies reported that pregnant women and/or women in the postnatal period were generally not satisfied with the quality of free maternal healthcare services provided
Assefa et al., 2019	Community health extension program of Ethiopia, 2003–2018: successes and challenges toward universal coverage for primary healthcare services	Systematic review	AFR	Effectiveness, safety, people centredness, equity, efficiency	Assess the successes and challenges faced by the community health extension programme in Ethiopia and develop a framework to strengthen the programme and progress toward universal coverage for primary healthcare services	Community health extension programme in Ethiopia has been associated with significant improvements in maternal and child health, communicable diseases, hygiene and sanitation, and knowledge and care seeking
Báscolo et al., 2018	Construction of a monitoring framework for universal health	Narrative review	AMR	Effectiveness, people centredness, timeliness, equity, efficiency	Develop a framework to monitor progress toward UHC	Identified 64 indicators for monitoring framework for universal health access and UHC, grouped under the following dimensions: strategic actions, outputs, outcomes and impacts
Bitton et al., 2019	Primary healthcare system performance in low-income and middle-income countries: a scoping review of the evidence base from 2010 to 2017	Scoping review	LMICs	Effectiveness, integrated care, people centredness, equity, efficiency, timeliness, safety	Assess the state of research on primary healthcare (PHC) in LMICs and identify priority areas for research	Highly researched areas included PHC policy, payment and workforce (including competence and motivation). Low research areas included population health management, facility management, effectiveness and quality of service delivery
Blanchet et al., 2012	How to achieve universal coverage of cataract surgical services in developing countries: lessons from systematic reviews of other services	Overview of systematic reviews	‘Developing’ countries	Effectiveness, timeliness, equity, integrated care, efficiency	Review evidence on effective strategies to promote coverage and access to eye care and other health services in ‘developing’ countries	No reviews met the study’s inclusion criteria for cataract surgery. Literature search pertaining to other health sectors identified several factors facilitating universal coverage in ‘developing’ countries including peer education, increased staff in rural areas, task shifting and integration of services
Boerma et al., 2014	Monitoring progress towards universal health coverage at country and global levels	Narrative review	Global	Effectiveness, timeliness, equity	Summarize evidence on monitoring progress toward UHC	Focusing on the levels of coverage and financial protection, with a focus on equity, in monitoring UHC is both relevant and feasible. UHC monitoring can be integrated into the monitoring of overall health system performance and progress
Bresick et al., 2019	Primary health care performance: a scoping review of the current state of measurement in Africa	Scoping review	AFR	Effectiveness, safety, people centredness, efficiency, timeliness	Summarize current state of measurement of primary care performance in Africa	Few validated instruments have been used to measure primary care performance in Africa. Further performance-based research is required to ensure access to high-quality care in a universal health coverage system
Christmals et al., 2020	Implementation of the National Health Insurance Scheme (NHIS) in Ghana: lessons for South Africa and low- and middle-income countries	Scoping review	AFR	Equity, people centredness, efficiency,	Synthesize evidence on the implementation of the NHIS in Ghana	Though NHIS has helped increase access to healthcare for the poor and most vulnerable, there are a number of challenges facing the NHIS, including poor perceived quality of care and ineffective governance
Fallah et al., 2021	Participation of delivering private hospital services in universal health coverage: a systematic scoping review of the developing countries’ evidence	Scoping review	'Developing' countries	Equity, efficiency,	Summarize evidence on the participation of private hospital services in advancing UHC in ‘developing’ countries	The role and contribution of private hospitals in efforts toward UHC differs depending on the country context
Farzaneh et al., 2020	The ethical framework for policy-making of universal health coverage: a systematic review	Systematic review	Global	Effectiveness, people centredness, equity, efficiency	Examine ethical frameworks used in the context of policy-making for UHC	Ethical frameworks used in UHC policy-making consist of ethical principles and criteria, including fairness, justice, sustainability, solidarity, good governance and efficiency
Gupta et al., 2018	Measuring progress toward universal health coverage: does the monitoring framework of Bangladesh need further improvement?	Systematic review	SEAR	Effectiveness, safety, equity	Compare Bangladesh’s monitoring framework for UHC to global-level recommendations proposed by WB/WHO and identify existing gaps in Bangladesh’s framework	Bangladesh’s UHC monitoring framework incorporates all of the global recommendations regarding financial risk protection and equity. However, there are significant gaps in indicators regarding service coverage in the areas of mental illness, cataract and neglected tropical diseases, despite a high disease burden attributable to these health conditions in Bangladesh
Hayati et al., 2018	Scoping literature review on the basic health benefit package and its determinant criteria	Scoping review	Global	Effectiveness, equity, safety, efficiency	Identify criteria used by countries globally to develop basic health benefit packages	The most widely applied criteria for basic health benefit packages globally are cost-effectiveness , effectiveness, budget impact , equity and burden of disease
Kamei et al., 2017	Toward advanced nursing practice along with people-centered care partnership model for sustainable universal health coverage and universal access to health	Narrative review	Global, WPR	People centredness, equity	Develop a people-centred care partnership model, to sustain UHC focused on ageing populations	Presented a people-centred care partnership model to address the health needs of an ageing society that centres the role of advanced practice nurses in sustaining UHC
Kiil, 2012	What characterises the privately insured in universal health care systems? A review of the empirical evidence	Systematic review	‘Developed’ countries	Equity, timeliness	Characterize patients who have voluntary private health insurance in UHC systems	Patients with voluntary private insurance in UHC systems have higher income and socioeconomic status. With a few exceptions, the privately insured are in equal or better health in comparison to the remaining population
Kim et al., 2020	Utilization of traditional medicine in primary health care in low-and middle-income countries: a systematic review	Systematic review	LMICs	People centredness, integrated care, timeliness, equity, effectiveness, safety	Examine the use and describe the strengths and limitations of traditional medicine in primary healthcare in LMICs	Traditional medicine is widely used in LMICs and helps increase access to healthcare, especially in low resource settings. However, some evidence demonstrates an association between traditional medicine and adverse health outcomes, including higher mortality. Further training of traditional medicine practitioners, and integrating their services within national health systems could help improve the quality of care provided
Lê et al., 2016	Can service integration work for universal health coverage? Evidence from around the globe	Systematic review	Global	People centredness, effectiveness, equity, efficiency, integrated care, timeliness	Assess the impacts of different types of service integration on service delivery, equity and health outcomes	Service integration can deliver incremental improved outcomes for both patients and healthcare providers without additional financial costs, with high levels of user satisfaction
Li et al., 2017	The development and impact of primary health care in China from 1949 to 2015: a focused review	Systematic review	WPR	Equity, effectiveness, safety, efficiency	Summarize the evidence on the development and impacts of PHC reforms in China and ongoing challenges	The Chinese government has focused on strengthening PHC, particularly after the SARS outbreak. Positive health outcomes have included reductions in child mortality and decreased maternal mortality rates. However, challenges remain including resource and workforce shortages, rural–urban disparities in health and inadequate utilization of PHC institutions, threatening the realization of ‘health for all’. Further investments and policy actions are required to improve China’s PHC system
Mate et al., 2013	Improving health system quality in low- and middle-income countries that are expanding health coverage: a framework for insurance	Narrative review	LMICs	Effectiveness, people centredness	Develop a framework to present insurance-driven strategies to improve quality of care within the context of UHC	A conceptual framework was created to present strategies available to public insurers responsible for expanding access to care, to influence healthcare quality. Framework further identified four mechanisms through which insurers can influence quality: investment in systems, patients and providers; selective contracting; provider payment; and benefit package design
McMichael et al., 2017	Health equity and migrants in the Greater Mekong Subregion	Scoping review	WPR, SEAR	Equity, people centredness	Examine the health needs of cross-border migrants in the Greater Mekong Subregion and their access barriers to healthcare and identify policy responses to improve their access to care	Despite increasing attention to migrant health globally, migrants continue to experience poor access to good quality care in the Greater Mekong Subregion due to legal, language and cultural barriers, as well as discrimination from healthcare providers. Further research is required to address the health needs of migrants in UHC efforts and advance health equity
Morgan et al., 2016	Performance of private sector health care: implications for universal health coverage	Narrative review	LMICs	Equity, effectiveness, efficiency, timeliness	Develop a conceptual framework that theorizes the linkages between private sector performance and wider health systems, and its implications for universal health coverage	The role of the private sector in supporting progress towards UHC in LMICs varies, and its performance is largely influenced by the characteristics of patients and providers, as well as the regulatory structures governing both the public and private sector. Influencing the performance of the private sector to benefit population health will require large-scale shifts that focus on the health system, as opposed to individual providers alone
Mumghamba et al., 2015	Capacity building and financing oral health in the African and Middle East region	Narrative review	AFR, EMR	Equity, efficiency	Summarize existing knowledge and identify gaps related to capacity building and financing of oral health in the African and Middle East region and identify priorities for future research	There is a lack of evidence on the impacts of oral health financing on the equity, efficiency and utilization of dental services in the African and Middle East region. Existing evidence suggests there are significant gaps between oral health needs and existing financial and human resource capacity. Further efforts are required to move toward universal coverage in oral health through innovative health insurance schemes and financing mechanisms
Naher et al., 2020	The influence of corruption and governance in the delivery of frontline health care services in the public sector: a scoping review of current and future prospects in low and middle-income countries of South and South-East Asia	Scoping review	SEAR, WPR	Equity, timeliness, efficiency, people centredness	Examine practices of corruption within PHC in the LMICs of the South and South-East Asia region and explore strategies to address these irregular and informal practices	Practices of corruption within health systems in the LMICs in the South and South-East Asia region are largely driven by poor governance and financial causes such as poor salary benefits and lack of adequate incentives. These practices increase out of pocket payments, reduce patient confidence in the health system and decrease utilization
Nandi et al., 2020	Using an equity-based framework for evaluating publicly funded health insurance programmes as an instrument of UHC in Chhattisgarh State, India	Narrative review	SEAR	Equity, effectiveness	Analyze the equity impacts of publicly funded health insurance (PFHI) schemes in Chhattisgarh State in India and identify evidence gaps	Evidence of high and equitable enrollment from household surveys may mask inequities within households among the most vulnerable. Equitable enrollment does not necessarily lead to financial protection or equity of utilization. Deepening inequities have been observed in utilization patterns as funds have been funnelled to better off areas and the private sector. The development of PFHI schemes, in the context of neoliberal policies that promote private sector provision of care, has significant consequences for health equity
O’Connell et al., 2015	Synthesizing qualitative and quantitative evidence on non-financial access barriers: implications for assessment at the district level	Systematic review	AFR, SEAR, WPR	Equity, people centredness	Examine non-financial barriers to access and utilization of maternal, newborn and child health services in Ghana, Bangladesh, Vietnam and Rwanda	Common non-financial barriers to access and utilization of maternal, newborn and child health services in Ghana, Bangladesh, Vietnam and Rwanda relate to ethnicity; religion; physical accessibility; decision-making, gender and autonomy; and knowledge, information and education
Palagyi et al., 2019	Organisation of primary health care in the Asia–Pacific region: developing a prioritised research agenda	Systematic review	SEAR, WPR	Efficiency, effectiveness, equity, integrated care	Identify evidence gaps and priority areas for future research related to evidence-based strategies for optimizing PHC service delivery in LMICs of the Asia–Pacific region	Five priority areas for future research are related to the optimal configuration of PHC teams; PHC service delivery management; task sharing/shifting; sustainable integration of PHC services; and equity-related outcomes
Palumbo, 2017	Keeping candles lit: the role of concierge medicine in the future of primary care	Systematic review	Global	People centredness, equity, effectiveness	Summarize evidence on the characteristics and effects of concierge medicine on UHC and sustainability of primary care services	Concierge medicine can lead to greater satisfaction among care providers and patients, generate additional revenue and increase the sustainability of the healthcare system. However, concierge practices are also likely to increase inequities in access to care and power imbalances between patients and providers
Petrou et al., 2018	Single-payer or a multipayer health system: a systematic literature review	Systematic review	Global	Equity, effectiveness, efficiency, timeliness	Examine the impacts of single payer and multipayer health systems on equity, efficiency, quality of care and financial protection globally	There is some evidence that single-payer systems are more equitable to patients than multipayer systems, which tend to be costlier due to higher administrative costs. In some cases, multipayer systems may be more efficient due to a lack of incentives for improvements to efficiency in single-payer systems
Ravaghi et al., 2018	A holistic view on implementing hospital autonomy reforms in developing countries: a systematic review	Systematic review	‘Developing’ countries	Equity, efficiency, effectiveness	Examine hospital autonomy reforms including their development, barriers and facilitators to implementation, their outcomes and implications for UHC in ‘developing’ countries	In general, hospital autonomy reforms in ‘developing’ countries have decreased financial protection, and increased inequities in access to quality health services, impeding progress toward UHC. Failure of these reforms can be attributed to a lack of a holistic, comprehensive view about what is required for success and poor/incomplete implementation
Rezapour et al., 2019	Developing Iranian primary health care quality framework: a national study	Narrative review	EMR	Equity, safety, effectiveness, people centredness, timeliness, efficiency	Create a framework to assess the quality of PHC within Iran’s health system	Literature review identified 13 Primary Health Care Quality Assessment Frameworks (PHCQAF), which evaluated the quality of PHC across 20 dimensions and 698 quality indicators. Delphi process resulted in the development of a PHCQAF for Iran, comprising 40 quality indicators across the dimensions of patient centredness; governance; access and equity; safety; efficiency and effectiveness. The largest share of indicators relates to the dimension of effectiveness (32.5%), while the lowest shares relate to dimensions of patient centredness, efficiency and governance (5% each)
Rodney et al., 2014	Achieving equity within universal health coverage: a narrative review of progress and resources for measuring success	Narrative review	Global	Equity, timeliness, efficiency	Examine how equity is conceptualized and measured within the context of UHC and describe strategies to assist decision-makers in implementing equity-enhancing UHC programmes	There is growing attention on the monitoring and evaluation of equity within UHC. Literature advocates for progressive universalism, in which the most disadvantaged are targeted in the planning of UHC programmes to advance equity. In efforts to monitor equity within UHC, countries should carefully assess the proposed WHO/WB framework prior to its adoption, as it focuses on wealth quintiles, and does not include other dimensions of equity such as gender and race, which could serve to mask increasing in-country disparities
Sanogo et al., 2019	Universal health coverage and facilitation of equitable access to care in Africa	Systematic review	AFR	Equity	Assess the effects of UHC on equitable access to care in Africa for vulnerable and underprivileged populations	In many African countries, efforts toward achieving UHC have increased access to care, but quality of care remains an ongoing issue, which disproportionately impacts the poor. Poor-quality care can lead to a lack of confidence in the health system and decrease utilization
Schmied et al., 2010	The nature and impact of collaboration and integrated service delivery for pregnant women, children and families	Narrative review	WPR	Integrated care, effectiveness, equity, people centredness	Examine the nature of collaboration and integration between care providers and the impacts of various forms of integration and collaboration for pregnant women, children and families	Various forms and degrees of collaboration and integration have been adopted in the delivery of universal health services. Well-coordinated or integrated services can positively impact the wellbeing of pregnant women, children, and families. Effective collaboration and integration require agencies and professional groups to overcome tension due to professional boundaries, break down cultural barriers and build trust
Schveitzer et al., 2016	Nursing challenges for universal health coverage: a systematic review	Systematic review	AMR	People centredness, integrated care	Summarize nursing challenges related to UHC	Nursing challenges related to UHC are due to gaps in education and training. A clearer definition of the nursing role in PHC is required
Sehngelia et al., 2016	Impact of healthcare reform on universal coverage in Georgia: a systematic review	Systematic review	EUR	Efficiency, equity, effectiveness	Assess the impacts of health system reforms in Georgia intended to ensure UHC on health financing sustainability, equity, efficiency, quality and cost control	Reforms implemented in Georgia to help ensure UHC have not been successful and have undermined health financing, efficiency, equity and the quality of care. Growth of privatization in the health sector without effective regulation and accreditation has hindered the quality of care
Sprockett, 2017	Review of quality assessment tools for family planning programmes in low- and middle-income countries	Narrative review	LMICs	Effectiveness, safety, people centredness, timeliness, equity, integrated care, efficiency	To identify quality assessment tools of relevance to clinic-based family planning programmes in LMICs	Identified 20 quality assessment tools of relevance to clinic-based family planning programmes in LMICs. A standardized quality assessment tool should be adopted to help achieve UHC, of which quality is a key component
Teerawattananon et al., 2016	How to meet the demand for good quality renal dialysis as part of universal health coverage in resource-limited settings?	Narrative review	EUR, WPR, SEAR	Safety, efficiency, effectiveness, people centredness, equity	Summarize the experiences of renal dialysis in seven study settings, describe how the quality of renal dialysis programs can be ensured, and discuss strategies to improve the quality of life of patients with end-stage renal disease	Five of the seven study settings have included renal dialysis as part of the UHC benefit package, with progress to do so in the remaining two settings. A holistic approach to disease prevention, identification and management, and appropriate use of financial mechanisms are required to ensure good-quality services and care for renal dialysis
Umeh, 2018	Challenges toward achieving universal health coverage in Ghana, Kenya, Nigeria, and Tanzania	Narrative review	AFR	Timeliness, equity, effectiveness, efficiency	Summarize the challenges to achieving UHC faced by Ghana, Kenya, Nigeria and Tanzania, and identify strategies to help ensure and strengthen UHC	Despite efforts to achieve UHC in many sub-Saharan African countries, significant challenges remain, including low informal sector enrollment and high rates of non-renewal of health insurance due to poor perceived quality of care
van Hees et al., 2019	Leaving no one behind? Social inclusion of health insurance in low- and middle-income countries: a systematic review	Systematic review	LMICs	Efficiency, effectiveness, equity	Assess the impacts of health insurance on vulnerable groups in LMICs	Unable to draw clear conclusions on the impacts of health insurance on financial risk protection, health outcomes and quality of care delivery for specific vulnerable groups in LMICs
Victora et al., 2004	Achieving universal coverage with health interventions	Narrative review	LICs	Effectiveness, equity, efficiency	Examine how known cost-effective health interventions in low-income countries can be taken to scale	Country specific strategies are required to scale up cost-effective interventions to reach the most vulnerable and reduce health inequities
White, 2015	Primary health care and public health: foundations of universal health systems	Narrative review	Global	Integrated care, equity, efficiency, effectiveness	Advocate for more integrated and universally accessible health services	Most health systems globally continue to focus heavily on illness. A renewed focus on public health and primary healthcare is essential to build sustainable health systems that are effective, efficient, equitable and affordable, and help realize the goals of UHC
Wiysonge et al., 2017	Financial arrangements for health systems in low-income countries: an overview of systematic reviews	Overview of systematic reviews	LICs	Equity, efficiency, effectiveness	Summarize evidence regarding the effects of financial arrangements for health systems in low-income countries	It is unclear whether financial incentives for health workers improve the quality of care provided by primary care physicians or outpatient referrals from primary to secondary care (very low-certainty evidence)
Yip et al., 2019	10 years of health-care reform in China: progress and gaps in universal health coverage	Narrative review	WPR	Efficiency, integrated care, effectiveness, safety, equity, timeliness	Assess whether health system reform efforts in China have succeeded in providing equal access to quality healthcare and financial risk protection	Health system reform efforts in China to advance UHC have resulted in mixed effects on quality. Issues related to provider competence remain, while many patients continue to be dissatisfied with the quality of care provided. However, there is some evidence of improved hospital performance in terms of process and outcome measures for some health conditions

CHW, community health worker; NHIS, National Health Insurance Scheme; NHS, National Health Service; UHC, universal health coverage; LMICs, low- and middle-income countries; PHC, primary healthcare; WPR, Western Pacific Region; AFR, African Region; AMR, Region of the Americas; EMR, Eastern Mediterranean Region; EUR, European Region; SEAR, South-East Asian Region; LICs, low-income countries

### Narrative synthesis of results

#### Conceptualizing universal healthcare/coverage and quality of care

The included studies highlighted varying definitions of UHC and quality of care. A common definition of UHC was that all people who require any essential healthcare services, including but not limited to promotion, prevention and treatment, are able to access services without financial stress [18–20]. One study further expanded this definition to include that UHC was the desired outcome of health system performance [18]. Some studies specified the definition was outlined in the Alma Ata declaration [21, 22].

Definitions of quality of care also varied. One study distinguished between service quality (e.g. patient satisfaction, responsiveness) and technical quality (e.g. adherence to clinical guidelines) [23]. Another study defined high-quality healthcare as ‘providing the highest possible level of health with the available resources’ [24, p. 142]. However, most studies did not provide a working definition of quality of care, and instead used proxy indicators such as infant mortality [25] to highlight quality-related outcomes.

#### *Synthesis according to Kruk et al. Conceptual framework*

Below, we synthesize findings from the studies according to the components of Kruk et al.’s [5] conceptual framework (foundations, processes of care and impacts). We highlight the most common themes that we identified in the literature for each domain and provide illustrative examples. Unless specified, findings were not specific to LMIC or HIC contexts.

### Foundations

#### Governance: leaders, policies, processes and procedures providing direction and oversight of health system(s)

A common theme across the literature was health system governance at local, regional and national scales, and its relationship to quality of care within the context of UHC. Naher et al. [26] identified transparency, accountability, laws and regulations, and citizen engagement as critical components of governance. The articles discussed both poor and good governance, their underlying determinants and drivers, as well as interventions to improve governance and thus quality of care [22–54].

The literature suggests that poor governance is a common issue across health systems, and is both a cause and indicator of poor-quality care. Causes and forms of poor governance include weak supervision of, and inadequate incentives and remuneration for healthcare providers; lack of transparency and accountability in decision-making; and insufficient financial capacity; in addition to fragmented regulations and policies. Poor governance has also been found to result in low patient trust and confidence in the health system, wasted resources and poor patient outcomes [26, 40, 44]. In contrast, the reviewed literature described strong governance as critical to effective healthcare services [26] and the basis for achieving UHC [32].

Interventions to improve governance described by the reviewed literature include decentralization, social accountability mechanisms, such as social audits, and policy reforms to strengthen provider incentives and service integration [26, 28, 31, 45, 47, 53]. However, the evidence regarding the effectiveness of these interventions on governance and quality of care was largely inconclusive. Regarding integration, White [45] noted the need to ensure adequate leadership and organizational capacity before integrating services, as a key determinant of success.

#### Quality of care measures

Six studies identified measures and/or measurement instruments to assess quality of care or its various dimensions within the context of UHC [19, 22, 27, 30, 42, 51]. These measures differed based on their service areas of focus (e.g. family planning, primary care), the geographic contexts for which they are intended and whether they assessed foundations, processes of care or quality impacts. The reviewed literature identified a lack of standardized quality assessment tools as a significant barrier to the realization of UHC [22, 42]. However, researchers also noted the need for country-specific indicators reflective of a country’s unique social, political and economic circumstances, and population needs and expectations [18, 22, 30, 39, 51﻿]. Studies also emphasized the importance of integrating equity as an explicit component in the measurement and monitoring of UHC through for example, disaggregation of data by key socioeconomic and demographic variables including place of residence, occupation, religion, ethnicity and migration status [18, 27, 30, 35]. Table 3 maps the measures identified in the studies according to the domains and subdomains of Kruk et al.’s framework.

**Table 3:** Quality domains and subdomains assessed by measures reported in the studies

Authors, year	Intended implementation context	Foundations					Processes of care		Quality impacts			Illustrative measures
		Populations	Governance	Platforms	Workforce	Tools	Competent care and systems	Positive user experience	Better health	Confidence in system	Economic benefit	
Agarwal et al., 2019	Monitoring framework to measure community health worker (CHW) performances in low- and middle-income countries		X	X	X	X	X	X		X		• Ratio of CHWs to supervisors • #/% of CHWs who have passed knowledge/competency tests (following training) • #/% of CHWs who correctly addressed (treated) the identified health problem (as per items in a checklist)
Báscolo et al., 2018	National-level monitoring framework for implementation in the Region of the Americas	X	X	X	X	X	X	X	X	X	X	• Density and distribution of health workers • Percentage of user satisfaction with the health services • Healthy life expectancy
Bresick et al., 2019	Primary care performance measurement in Africa		X		X	X	X	X		X		• Identifies eight validated instruments to measure primary care performance in Africa. No specific measures reported
Gupta et al., 2018	National-level UHC monitoring framework for Bangladesh	X		X			X		X	X	X	• Tuberculosis treatment success rate • Case fatality rate among hospitalized acute respiratory infection cases
Rezapour et al., 2019	Iranian Primary Health Care Quality Assessment Framework	X	X	X			X	X	X	X		• % of safe injections in the healthcare facility • Customer satisfaction rate (%) • % of patients aware about patients’ rights and responsibilities
Sprockett, 2017	Quality assessment tools for family planning programmes in low- and middle-income countries		X	X	X	X	X	X				• No specific measures reported

#### Skills and availability of health system workers

Several studies also identified critical health workforce shortages and inequities in the distribution of appropriately qualified staff between urban and rural areas as significant constraints to the provision of high-quality, equitable care within the context of UHC, particularly in LMIC contexts [21, 23, 25, 29, 31, 38, 40, 43, 44, 46–50, 53]. Strategies discussed to address these concerns included (i) improving recruitment and retention of health system staff for rural and remote areas [21, 46, 47, 50]; (ii) recruiting and training community health workers, while increasing the skills of lay health workers [21]; (iii) training traditional medicine practitioners in conventional medicine and utilizing them as community health workers [49]; and (iv) increasing task shifting, through delegating tasks to less specialized health workers [21, 31], for which supportive supervision and adequate training is required [21].

### Processes of care

#### Access to competent care and systems, incentives to improve quality of care delivery

Evidence from the reviewed studies suggests that poor provider competence across a range of health services remains an ongoing issue, particularly in LMICs, posing a considerable barrier to the provision of timely, safe and effective quality of care [22, 23, 29, 31, 33, 39, 40, 46, 47, 49]. For example, in China, a study with standardized patients found that providers in village hospitals provided correct treatment for tuberculosis only 28% of the time [47].

Within health systems seeking to provide UHC, significant inequities remain in both LMICs and HICs regarding the quality of care received by different populations. Vulnerable populations, who are more likely to receive care from lower-level health facilities, such as health centres, are disproportionately impacted by incompetent care and systems, having already constrained access to care [26], fewer options regarding providers and being more likely to receive inappropriate referrals [40], all indicators of lower-quality care.

Four studies described organizational factors influencing provider competence, including performance appraisal, continuing education, incentives, and remuneration and payment mechanisms [27, 31, 40, 46]. For example, Sanogo et al. [40] discussed how delays in provider reimbursement as observed in Ghana, can demotivate healthcare providers, which Agarwal et al. [27] noted may decrease providers’ willingness to exert maximum effort on assigned tasks, compromising the quality of care.

Regarding incentives to improve motivation and quality of care delivery, Yip et al. [47] suggested a pay-for-performance system in China, as physicians are traditionally incentivized for treatment-based care through fee-for-service. However, the systematic review from Wiysonge et al. [46] noted a lack of evidence to support whether financial incentives for healthcare providers would improve quality of care in low-income countries.

#### User experience: wait times and people centredness

Wait times, a core component of quality of care, were noted as ongoing concerns in HICs and LMICs [21, 23, 33, 39, 40, 47, 48, 55, 56]. In HICs such as Norway and the United Kingdom, long wait times have been found to increase the demand for duplicative voluntary private health insurance, which Kiil argues may threaten the overall quality of public-sector driven UHC and exacerbate inequities [56]. In LMICs, evidence has shown that service quality is often superior in the private sector compared with the public sector, defined in relation to shorter wait times, better hospitality and increased time spent with providers [23].

Several studies described the relationship between positive user experience and people-centred care, which focuses on the needs and preferences of populations served while engaging them in shaping health policies and services. In addition, people centredness has been linked to improved mental and physical health, and reduced health inequities among other outcomes [20, 22, 31, 35, 57].

One study presented a people-centred care partnership model intended to support the work of advanced practice nurses in sustaining UHC, identifying nine attributes of people centredness including mutual trust and shared decision-making [20].

Several studies also discussed strategies aimed at increasing patient/community voice and engagement and the people centredness of health systems. These strategies included citizenship endorsement groups in Mexico [34] and various public forums to foster accountability and transparency [26]. However, McMichael et al. [35] cautioned that approaches to increase the voice of patients and communities risk excluding the most vulnerable, as those facing the greatest barriers to participation in such initiatives are often the most disadvantaged in their access and use of health services.

### Quality impacts

#### Quality of care outcomes

A few of the reviewed articles reported on empirical studies that analyzed patient and population health outcomes in relation to quality of care in the context of UHC. Where reported, these outcomes were discussed in reference to (i) specific programmes intended to improve quality of care and advance UHC, (ii) the impacts of health insurance schemes or health system reforms, (iii) private versus public sector provision of healthcare and/or (iv) the effects of specific service delivery models.

(i) Regarding programmes intended to improve the quality of care, a community health extension programme in Ethiopia was associated with increased perinatal survival and decreased prevalence of communicable diseases. Though resource constraints such as inadequate medical supplies and limited supervision of health extension workers were noted as challenges, a key success factor included strong community engagement [29].
(ii) Another six studies examined health outcomes in relation to health insurance schemes or health system reforms [25, 40, 46–48, 55]. Some improvements in health outcomes were noted. For example, in China, health system reforms aimed at achieving UHC have been associated with decreased maternal mortality rates [25]. However, the burden of noncommunicable diseases such as diabetes is rising amid significant gaps in their detection and treatment [47].
(iii) Studies also compared patient outcomes in relation to private versus public sector healthcare provision [24, 56, 58]. How the private sector was conceptualized varied across the studies, both in terms of how it was categorized (e.g. for-profit versus not-for-profit), as well as its role in healthcare financing and delivery. Given this heterogeneity, whether the public or private sector leads to higher-quality care and consequently, better health outcomes, is unclear in the reviewed literature. However, the private sector, when financed through out-of-pocket payments, is more likely to exacerbate inequities in access to healthcare.
(iv) Finally, two studies examined integrated models of care and their relationship to health outcomes [52, 54]. According to these studies, different forms of service integration may positively impact health, for example, through slowed disease progression [54] and decreased preterm births [52].

#### Patient-reported satisfaction and trust in health system

Reports of poor perceived quality of care and low patient satisfaction as barriers to healthcare uptake and enrollment in health insurance schemes were common across the reviewed studies [26, 28, 36, 40, 44, 47, 55, 56]. For instance, Alhassan et al. [28] found that perceived low quality of care, long wait times and poor treatment by healthcare providers reduced clients’ trust in Ghana’s National Health Insurance Scheme, reducing subsequent re-enrollment rates. In Ghana, perceived quality of care was found to exert a greater influence on men’s decisions regarding care uptake than on women’s decisions [36, 44]. O’Connell et al. [36] suggested this gendered difference may be due to men’s care being more likely to be prioritized within household financial decisions, affording them the opportunity to be more discerning regarding the quality of care.

Several studies also discussed the effects of health system reforms and different service delivery models on patient satisfaction and trust in healthcare systems [23, 28, 29, 31, 38, 43, 47, 54, 57]. Yip et al. noted that despite reforms aimed at expanding access to care across China, many patients have chosen to forgo care at primary healthcare facilities altogether due to a lack of trust and dissatisfaction with quality of care [47]. Similarly, Ravaghi et al. identified contradictory results regarding the effects of hospital autonomy reforms on patient satisfaction. Two studies in Indonesia cited in Ravaghi’s review reported improvements, while others noted decreased or no change in patient satisfaction [38]. In contrast, four reviews found that integrated, people-centred health services may positively impact patient satisfaction [29, 31, 54, 57].

#### Efficiency of healthcare services and systems

Twenty-seven studies addressed the efficiency of healthcare systems and services, which the review by Morgan et al., defined as ‘the extent to which resources are used effectively or are wasted’ [23, p. 608]. These studies discussed inefficiencies in health systems [22, 26, 28, 29, 44, 48], the possible effects of health reforms and other interventions on efficiency [21, 25, 31, 37, 38, 41, 44–47, 50, 53–55, 58, 59], efficiency as a criterion in health policymaking [32], and the measurement of efficiency [22, 30, 42, 51], an example of which, as cited in Rezapour et al.’s study, was the percentage of prescriptions including antibiotics in health centres and health posts [51].

Additionally, some studies compared the efficiency of public and private sector healthcare provision, reporting mixed results [23, 24, 48, 58, 61]. For example, higher overhead costs and lower quality of care outcomes, including higher death rates, have been observed in private hospitals compared with public hospitals in the United States [24]. In contrast, research on the National Health Service in England has suggested that privatization and market-oriented reforms have improved the efficiency of hospital care through cost cutting without evidence of reduced quality [58].

In LMICs, the private sector has been linked to increased service costs related to overprescribing and use of unnecessary and expensive procedures [23]. However, Morgan et al. noted that studies assessing private sector performance in LMICs have often focused on unqualified or informal small private providers, such as small drug shops, operating amid weak public health systems and poor regulation, providing an incomplete picture of the role of the private sector in progress towards UHC [23]. Table 4 captures a high-level overview of the key highlights related to each domain and subdomain of Kruk et al.’s [5] framework discussed in the studies.

**Table 4:**
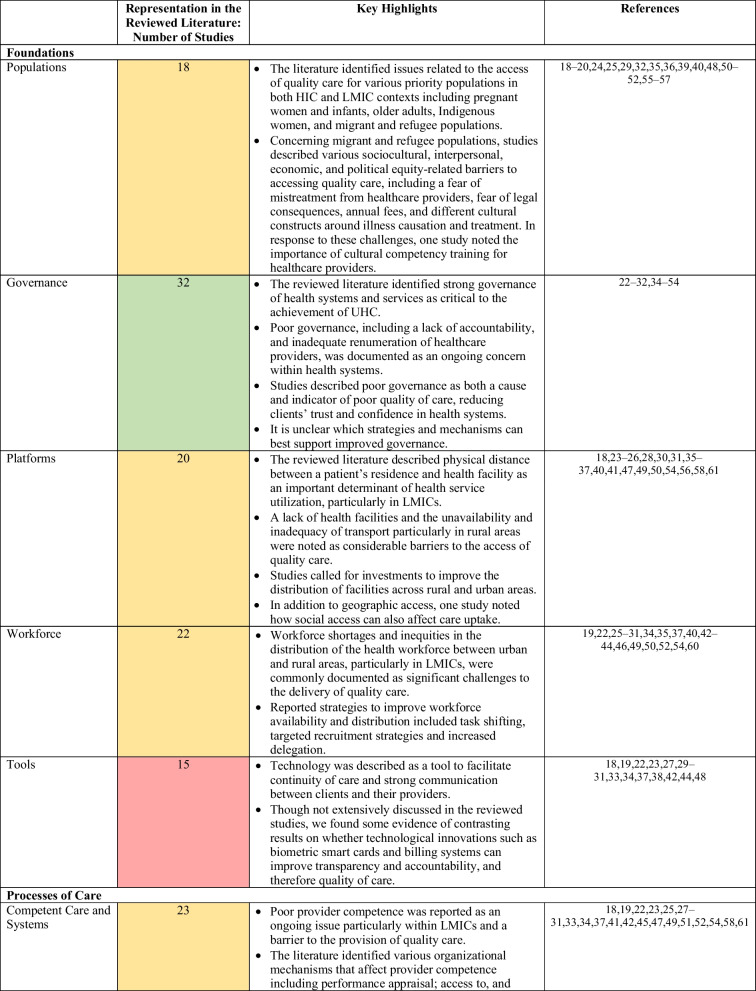

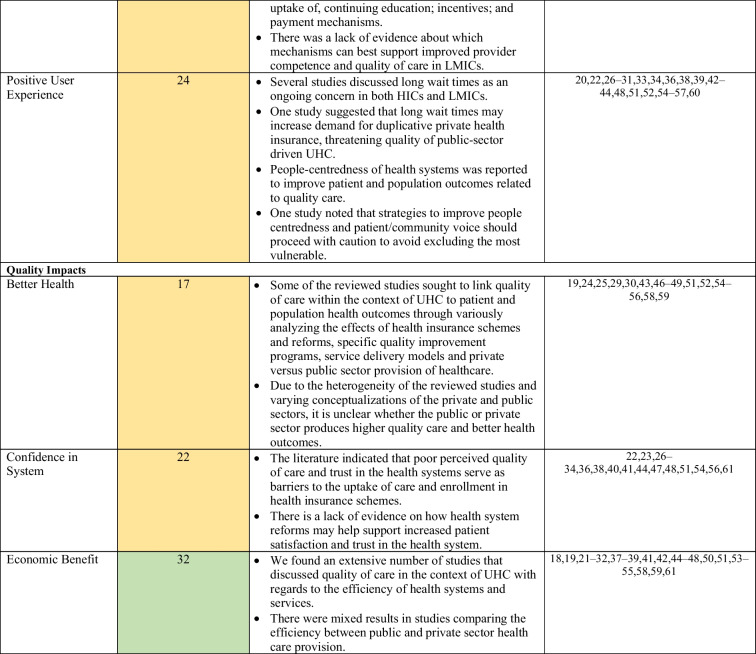
Overview of key findings mapped to the domains and subdomains of Kruk et al.’s framework

In the middle column, cells are shaded according to the representation of the (sub)domain in the reviewed literature. Green = high representation (30–45 studies), yellow = moderate representation (16–29 studies), red = low representation (0–15 studies)

### Identified evidence gaps and priorities for future research

Substantial evidence gaps that were identified in the reviewed literature are grouped thematically below. Themes are ordered by how frequently they were discussed by the reviewed studies.

### Gap 1: How to measure and monitor UHC, with particular attention to quality of care and equity

Several studies identified the need for additional research to inform the development, selection and use of monitoring and evaluation frameworks and measures to assess quality of care and equity in relation to UHC in various geographic contexts at multiple levels of the health system, including facility and institutional levels [22, 30, 31, 34, 39, 42]. For example, Rodney et al. stressed that countries should select contextually relevant indicators, and pay particular attention to the measurement of equity within UHC, cautioning that measuring equity based solely on wealth quintiles may mask inequities related to other factors such as race or disability [39]. In addition, two studies discussed the lack of client-reported measurements and advocated for further research to integrate data from household surveys and user-experience surveys [22, 30].

### Gap 2: Comparative information on the efficiency and effectiveness of public and private health provision and appropriate mix of public and private healthcare

Researchers noted the need for more conclusive evidence comparing the efficiency and effectiveness of public and private health sector provision, and the role of the private sector in contributing to UHC [21, 23, 56, 57, 62]. For example, Morgan et al. highlighted the need for greater evidence on how system-level influences such as regulations, may be used to create a public–private healthcare mix that promotes high-quality care and supports the achievement of UHC [23].

### Gap 3: Effects of financial and insurance schemes on quality-of-care delivery and patient outcomes

The reviewed literature identified a lack of evidence regarding the impacts of different financial and insurance schemes on quality-of-care delivery and patient outcomes, particularly for vulnerable groups including women-headed households, children with special needs and migrants [34, 46, 55, 62]. For example, van Hees et al. noted a lack of evidence regarding the impacts of financial schemes, such as pooling of funds and cost sharing, on equity [55].

### Gap 4: Effects of integrated service delivery models

Studies identified the need for more robust evidence related to the effects of integrated service delivery models on access to quality care, as well as patient and population health outcomes [22, 37, 52, 54]. Lê et al. specifically highlighted the lack of evidence on equity outcomes related to service integration, suggesting the need for further research in this area [54].

### Gap 5: Mechanisms and contexts that enable and hinder implementation of quality-related interventions

Finally, researchers called for additional evidence regarding the mechanisms and contextual factors such as societal stigma that influence the effectiveness of interventions related to quality of care in the context of UHC [34, 37, 55]. To this aim, van Hees et al. recommended realist evaluations to surface what works, for whom, and in what contextual circumstances [55]. For example, Palagyi et al. identified a need for further research on task shifting, particularly how the skills gained by health workers can be maintained, and its implications for team dynamics and the delivery of existing programmes [37].

## Discussion

This scoping review aimed to characterize the existing conceptual and empirical literature on quality of care within the context of UHC. As noted in our results, in the reviewed literature, quality of care was often ill defined or defined inconsistently. A lack of conceptual clarity compromises the development of a robust evidence base able to inform the design and implementation of effective quality-related policies and interventions.

The 45 articles we reviewed for our study reveal a heterogeneous body of literature when compared with Kruk et al.’s quality of care framework. While some framework components including governance and the efficiency of healthcare services and systems were highly represented in the included literature, others were less represented such as physical and technological resources and tools, and patient and population health outcomes.

We also noted in the reviewed literature a lack of clarity regarding how the studies distinguished between private sector involvement in financing and/or delivery of care. This lack of clarity limits our understanding of the implications of private sector engagement for the quality of care and the achievement of UHC in various geographical contexts. Research is required to provide greater clarity of the role and impacts of private sector involvement in financing and/or delivery of health services, to help inform countries’ decision-making regarding private sector engagement. In addition, further research is needed regarding the interactions between the public and private sector and their effects on the sustainability of UHC. For example, studies have noted a concern that the availability of concierge services can create downstream implications for people who cannot afford private insurance, such as an imbalance in resource distribution [57].

Overall, the identified evidence gaps pointed to the need to build a stronger evidence base about what works, for whom, and under what contextual circumstances, and with what effects on equity to improve quality of care in LMICs and HICs. This includes a need for further evidence on the effects of integrated service delivery models, as well as how regulation can be used to create a public–private healthcare mix promoting high-quality and equitable care. The literature further highlighted the urgent need for additional research to inform the creation of robust monitoring and evaluation frameworks prioritizing equity that could support improvements to quality of care. This includes further research to help support the inclusion and use of disaggregated data, such as by wealth, sex and ethnicity to monitor and inform efforts to increase equity in access, utilization and outcomes for vulnerable populations. Beyond the above-noted research priorities, we also recommend additional research comparing quality related outcomes before and after UHC implementation, and how they intersect with health equity.

Strengths of our scoping review include the use of a broad search methodology and validated search filters in consultation with an expert librarian, and the use of a conceptual framework to guide analysis of findings. Further, our search was not constrained based on country of origin. In our search of the literature, we did not find other published reviews of similar scope about quality of care within the context of UHC.

The primary limitation of our review is the small number of included studies that met our eligibility criteria. This highlights that quality-related research in UHC remains an emerging field. In addition, many of the included studies were narrative reviews, which may not have captured the full breadth of the literature. Another limitation of our review is that we included only English-language studies. Future reviews should attempt to search and synthesize evidence in additional languages to provide more global relevance. Further, the conceptual framework we applied to the analysis of findings does not consider various factors that render health systems more fragile such as pandemics, disasters and conflicts, which may compromise the quality of care and realization of UHC. As our study did not include search terms for specific vulnerable populations such as Indigenous or racialized groups, there is also need for future research related to LMICs and communities experiencing marginalization and discrimination within HICs.

In addition, there may be limited applicability of findings across studies to different geographic regions. Finally, due to the heterogeneity and qualitative nature of the included studies, meta-analysis and synthesis beyond thematic analysis were not feasible.

## Conclusion

This review summarized the existence of available evidence on quality of care within the context of UHC, identifying strategies aimed at improving quality of care as well as diverse knowledge gaps. Further research, evaluation and monitoring frameworks including those that attend to equity are required to strengthen the existing evidence base.

## Data Availability

This work analyzed secondary sources, which are cited and are publicly accessible or with academic institutional credentials. Authors can confirm that all other relevant data are included in the article and/or its additional files.

## APPENDIX A: Appendix: Search Strategy

### Database: Ovid MEDLINE: Epub Ahead of Print, In-Process & Other Non-Indexed Citations, Ovid MEDLINE® Daily and Ovid MEDLINE®

#### 1946: September 27, 2021

**Table:**

#	Searches	Results
1	Universal health insurance.mp or exp Universal Health Insurance/	4022
2	(UHC or (universal adj2 (coverage or care or healthcare or healthcare or health-care)) or ((universal or population or public) adj2 (healthcare or health care or health-care or health coverage or healthcare coverage or health care coverage or health-care coverage or access to care or access to health or access to healthcare or access to health care or access to health-care or access to health service* or access to medicine* or health access or healthcare access or health care access or health-care access or health service* access or medicine* access or health insurance or healthcare insurance or health care insurance or health-care insurance))).mp	34,774
3	exp Quality Improvement/ or exp Quality Indicators, Health Care/	43,714
4	quality.mp	1,170,848
5	((integrat* adj2 care) or (consult* or participat* or collab* or partner*) or ((people or person) adj2 cent*) or effective* or timel* or safe* or efficien*).mp	4,263,417
6	(((systematic OR state-of-the-art OR scoping OR literature OR umbrella) ADJ (review* OR overview* OR assessment*)) OR "review* of reviews" OR meta-analy* OR metaanaly* OR ((systematic OR evidence) ADJ1 assess*) OR "research evidence" OR metasynthe* OR meta-synthe*).tw. OR exp Review Literature as Topic/ OR exp Review/ OR Meta-Analysis as Topic/ OR Meta-Analysis/ OR "systematic review"/	2,838,112
7	1 OR 2	34,774
8	3 OR 4 OR 5	5,042,750
9	6 AND 7 AND 8	1799
10	limit 9 to yr = "1995 -Current"	1612

## Abbreviations

AFR: African Region
AMR: Region of the Americas
CHW: Community Health Worker
EMR: Eastern Mediterranean Region
EUR: European Region
HICs: High-Income Countries
LMICs: Low- and Middle-Income Countries
MDGs: Millennium Development Goals
NHIS: National Health Insurance Scheme
NHS: National Health Service
PHC: Primary Healthcare
PRISMA: Preferred Reporting Items for Systematic Reviews and Meta-Analyses
SDGs: Sustainable Development Goals
SEAR: South-East Asian Region
UHC: Universal Health Coverage
WPR: Western Pacific Region
